# Correction: *In silico* decryption of serotonin–receptor binding: local non-covalent interactions and long-range conformational changes

**DOI:** 10.1039/d1ra90088a

**Published:** 2021-03-08

**Authors:** Padmabati Mondal

**Affiliations:** Department of Chemistry and Center for Atomic, Molecular and Optical Sciences and Technologies, Indian Institute of Science Education and Research (IISER) Tirupati Tirupati 517507 Andhra Pradesh India padmabati.mondal@iisertirupati.ac.in +91 877 2500 926

## Abstract

Correction for ‘*In silico* decryption of serotonin–receptor binding: local non-covalent interactions and long-range conformational changes’ by Padmabati Mondal *et al.*, *RSC Adv.*, 2020, **10**, 37995–38003, DOI: 10.1039/D0RA05559J.

In the original article, the BRIL fusion in the crystal structure was mistakenly understood as mini-G_0_. All simulations in the original article were based on the structure of serotonin–receptor with BRIL fusion (OB1). Hence, all ‘mini-G_0_’ in the article (in text and figures) should be read as ‘OB1’. As OB1 is not a part of the G-protein, the long-range conformational connection between receptor and mini-G_0_ regarding the signal transduction of serotonin–receptor *via* the mini-G_0_ region of the G-protein, cannot be concluded as described in the article. In the following, long-range conformational changes due to serotonin binding to the receptor coupled to mini-G_0_ is discussed. The initial structure of the receptor (5HT_1*B*_) coupled to mini-G_0_ is taken from PDB ID: 6G79.^1^ A serotonin molecule is docked to the receptor (following the same procedure as described in the original article) to obtain the serotonin–receptor complex (Fig. 1). For the simulation and analysis, exactly the same procedure as discussed in the original article is followed. Since the receptor protein and serotonin binding pocket remain invariant, the local non-covalent interactions between serotonin and the receptor remain invariant too, and this is also supported by previous literature.^1^ The structure of serotonin bound 5HT_1*B*_ coupled to mini-G_0_ is shown in Fig. 1. The C-terminal amino acid Tyr354 from mini-G_0_ couples to the 5HT_1*B*_ receptor *via* stacking interactions as well as weak polar interactions.^1^

**Fig. 1:**
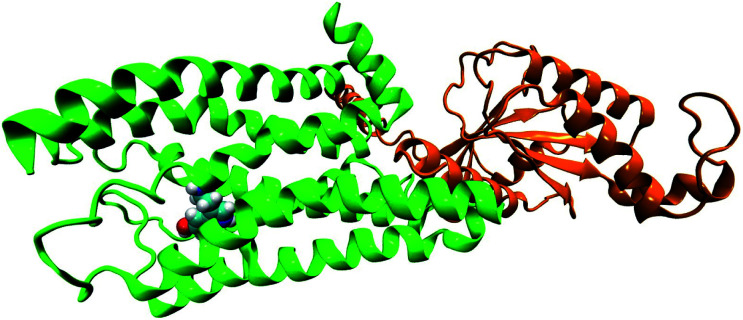
The structure of G-protein coupled receptor 5HT_1*B*_ (green) bound to serotonin (in VDW representation) along with mini-G_0_ (orange).

Fig. 2a shows the comparison of 2D projections of the eigenvector 1 and eigenvector 2 corresponding to the changes in the two most important collective motions for the apo-receptor (black) and the complex (red). The non-overlapping region between the red and black dots accounts for the conformational changes due to serotonin–receptor binding. In order to identify the important region contributing to the conformational stability/instability due to the serotonin–receptor binding, the root-mean-square fluctuations (RMSF) of the C_*α*_ of each residue for the first five important collective motions are plotted (in Fig. 2b) for the apo-receptor (black) and the complex (red) along the residue numbers.

**Fig. 2:**
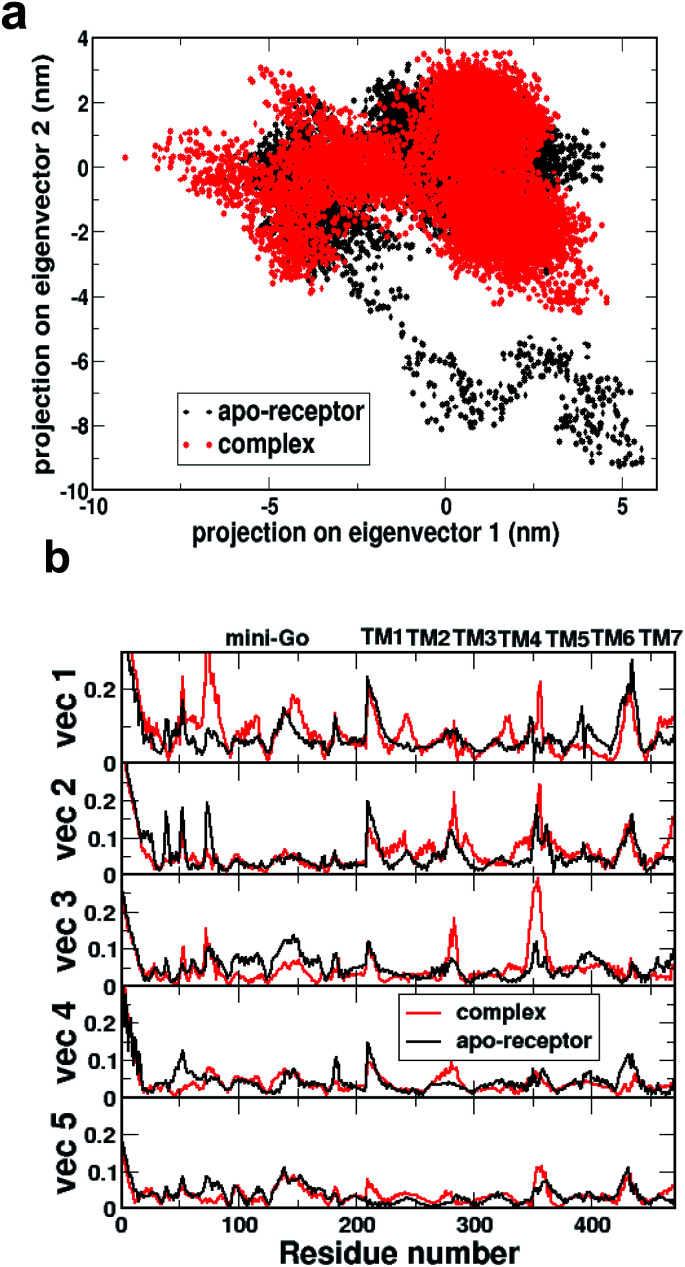
(a) Comparison of 2D projection of the two eigenvectors (eigenvector 1 on eigenvector 2) corresponding to the most important conformational changes of the receptor for apo-receptor (black dots) and holoreceptor or complex (red dots). (b) Comparison of root-mean-square fluctuations (in nm) of each residue based on the eigenvectors corresponding to the first five most important conformational changes along the residue numbers for the apo-receptor (black) and the complex (red).

Fig. 2b shows that the RMSF for residue number 0–200 (mini-G_0_) is significantly increased for the complex for vec1 for the complex with respect to the apo-receptor, whereas the RMSF is reduced for vec2, vec3, vec4 and vec5. On the other hand, for the receptor part, while the RMSF for TM5 and TM6 is reduced due to serotonin binding, the RMSF for TM1, TM2, TM3, TM4 and TM7 is increased for the complex with respect to the apo-receptor. This indicates that although serotonin binding stabilizes local helices (*e.g.* TM6, TM5) due to non-covalent interactions, the other part of the receptor as well as the mini-G_0_ region, encounter instability due to the allosteric effect. This in turn contributes to the conformational changes in the mini-G_0_ region of the G-protein due to serotonin binding and very likely helps in the signal transduction through G-protein.

Fig. 3 shows the difference dynamic cross correlation (DDCC) map where the difference between the DCC map for the serotonin–receptor complex and apo-receptor are plotted with a color map in the range of [−0.8 : 0.8] for the C_α_ of the receptor from 10 000 snapshots (in 10 ps interval) from 100 ns simulation. The figure indicates that the correlation between TM1 and mini-G_0_, TM3 and mini-G_0_, TM4 and mini-G_0_ as well as TM6 and mini-G_0_, are different in the complex with respect to the same in the apo-receptor. It should be noted here that the TM6 region contains Phe330, Phe331 and Trp327 which are the three most important aromatic residues, having stacking interactions with the serotonin. On the other hand, the TM3 region contains Asp129 which has strong hydrogen bonding with the amine group of serotonin. Within the receptor, the conformational correlations of TM4 and TM6 with all other regions become stronger due to serotonin binding.

**Fig. 3:**
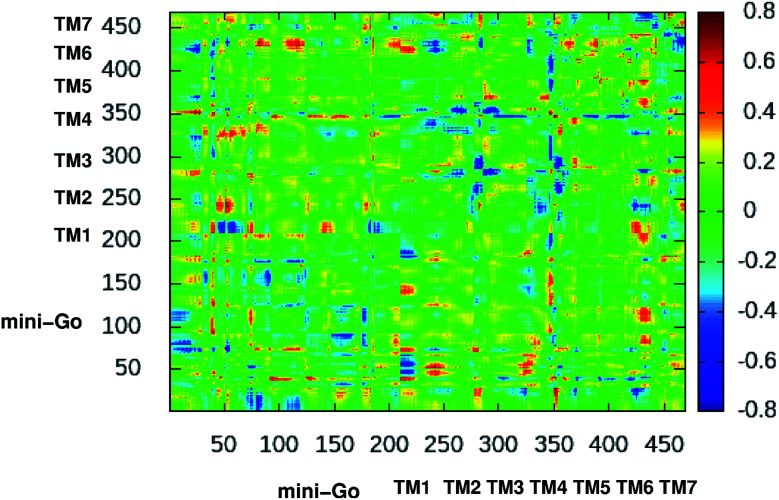
2-Dimensional difference dynamic cross correlation (DDCC) map with color map in the range [−0.8 : 0.8], calculated as per the method described in the original manuscript. The color scale is shown on the right.

In summary, the results for long-range conformational changes upon serotonin binding discussed in the original article (with OB1) is comparable to what is described here (with mini-G_0_) except for a few subtle differences as described above. Therefore, it can be concluded that the inference regarding the allosteric effect on the mini-G_0_ due to serotonin binding, which may help in signal transduction, is still valid.

The Royal Society of Chemistry apologises for these errors and any consequent inconvenience to authors and readers.

## Supplementary Material
